# Sediment-associated microbial community profiling: sample pre-processing through sequential membrane filtration for 16S rRNA amplicon sequencing

**DOI:** 10.1186/s12866-022-02441-0

**Published:** 2022-01-20

**Authors:** Joeselle M. Serrana, Kozo Watanabe

**Affiliations:** Center for Marine Environmental Studies (CMES), Ehime University, Bunkyo-cho 3, Matsuyama, Ehime, 790-8577 Japan

**Keywords:** Sediment-associated microbial communities, River sediments, Sequential membrane filtration, 16S rRNA amplicon sequencing

## Abstract

**Background:**

Sequential membrane filtration as a pre-processing step for capturing sediment-associated microorganisms could provide good quality and integrity DNA that can be preserved and kept at ambient temperatures before community profiling through culture-independent molecular techniques. However, the effects of sample pre-processing via filtration on DNA-based profiling of sediment-associated microbial community diversity and composition are poorly understood. Specifically, the influences of pre-processing on the quality and quantity of extracted DNA, high-throughput DNA sequencing reads, and detected microbial taxa need further evaluation.

**Results:**

We assessed the impact of pre-processing freshwater sediment samples by sequential membrane filtration (from 10, 5 to 0.22 μm pore size) for 16S rRNA-based community profiling of sediment-associated microorganisms. Specifically, we examined if there would be method-driven differences between non- and pre-processed sediment samples regarding the quality and quantity of extracted DNA, PCR amplicon, resulting high-throughput sequencing reads, microbial diversity, and community composition. We found no significant difference in the qualities and quantities of extracted DNA and PCR amplicons, and the read abundance after bioinformatics processing (i.e., denoising and chimeric-read filtering steps) between the two methods. Although the non- and pre-processed sediment samples had more unique than shared amplicon sequence variants (ASVs), we report that their shared ASVs accounted for 74% of both methods’ absolute read abundance. More so, at the genus level, the final collection filter identified most of the genera (95% of the reads) captured from the non-processed samples, with a total of 51 false-negative (2%) and 59 false-positive genera (3%). We demonstrate that while there were differences in shared and unique taxa, both methods revealed comparable microbial diversity and community composition.

**Conclusions:**

Our observations highlight the feasibility of pre-processing sediment samples for community analysis and the need to further assess sampling strategies to help conceptualize appropriate study designs for sediment-associated microbial community profiling.

**Supplementary Information:**

The online version contains supplementary material available at 10.1186/s12866-022-02441-0.

## Background

Microorganisms have long been recognized as valuable bioindicators for biomonitoring and ecological assessment of freshwater ecosystems [1–3]. Recent studies took advantage of high-throughput sequencing (HTS) to characterize freshwater sediment-associated microorganisms for impact assessment of anthropogenic activities and environmental factors on diversity and composition, and their functions [e.g.,^4^, ^5^]. In particular, 16S rRNA amplicon sequencing is a relatively faster and cheaper approach providing substantially higher taxonomic resolution [6], with the capability of detecting unculturable, rare, and novel microorganisms [7] in comparison to the conventional strategies, e.g., culture-dependent methods [8], and other molecular approaches, e.g., shotgun metagenomics and metatranscriptomics for community profiling.

The characterization of microbial communities from environmental sediment samples usually involves the direct extraction of DNA, amplification of a target region, i.e., the hypervariable region of the 16S rRNA gene, through polymerase chain reaction (PCR), amplicon library construction, and sequencing on a high-throughput platform (e.g., Illumina-based technologies). One major challenge is the isolation and capture of good quality and quantity DNA from sediment samples [9, 10], which mainly contains impurities that inhibit PCR amplification [11]. Various commercial extraction kits are available for the rapid processing of environmental samples tailored to yield abundant and high-quality DNA minimizing the effects of enzyme inhibitors, e.g., humic acid, polysaccharides, metals, etc., that must be removed before amplification with the help of proprietary chemicals [12–14]. However, most of these kits commonly rely on DNA-binding steps via silica spin columns for DNA purification and concentration. This procedure possibly results in DNA loss due to competitive column-binding of organic matter [15], which may also selectively retain high molecular-weight DNA fragments [16].

Pre-processing sediment samples by multi-level or sequential membrane filtration have been reported to efficiently isolate high-quality DNA while reducing inhibitory compounds [10, 17–19]. Sequential filtration has been used to concentrate microbial biomass and assess communities based on size fractions using filter membranes with different pore sizes [20, 21]. A pre-filter of larger pore size (1.0 to 30 μm) and a collection filter of smaller size (0.22 μm) are commonly used in-line series of filters [22–25] to efficiently capture viruses, bacteria, and parasites based on size exclusion [26, 27]. DNA is then extracted from the final collection filter to separate targeted microorganisms from the comparatively larger eukaryotic cells [e.g., 28] or remove large particle-associated microbes from the free-living fraction [e.g., 20, 29, 30,31,32 33]. However, pre-processing sediment samples is not commonly practiced because it is relatively more laborious than directly processing the sediments, and that its pros and cons in comparison to the standard method requires further assessment.

Previous studies have characterized and compared the microbial community structure of various collection strategies against in situ*,* or on-site filtration of particle or sediment collected samples, mainly from marine environments [e.g.,^34^, ^35^]. On-site filtration keeps the sampled microbial communities in situ conditions while reducing collection and storage time [34]. The microorganisms from environmental samples should be inactivated right after collection without significant damage to their DNA [36]. Managing this time is critical to prevent bacterial overgrowth or taxonomically biased DNA damage and degradation [37]. Integrating filtration as a pre-processing step for capturing microorganisms could provide good quality and integrity DNA from sediment samples that can be preserved sufficiently well and kept at ambient temperatures before DNA extraction and library construction for HTS-analyses. Most of the studies on applying pre-processing sediment samples by sequential membrane filtration focused on the quality assessment and efficiency of the extracted metagenomic DNA. Solomon et al. [10] demonstrated that community DNA with minimal shearing was obtained from pre-processing marine sediment samples against non-processed and performed PCR amplification of the 16S rRNA gene to confirm that the filtration method isolated high-quality DNA. A similar protocol was employed to process arctic sediment samples to characterize bacterial community structure by 16S rRNA amplicon sequencing [17]. However, there is no comprehensive information on sequential membrane filtration's potential biases on the retained microbial taxa than its non-processed counterpart, specifically whether sample pre-processing via sequential filtration compares to non-processed community profiles for quantitative measurements of freshwater microbial diversity and community structure.

Here, we examined if there would be method-driven differences between non- and pre-processed sediment samples (represented by the collection filter) by sequential membrane filtration for microbial community profiling through 16S rRNA amplicon sequencing. Very coarse sand and gravel sediment samples collected from selected gravel bars in a dam-impounded river were used in this study. Specifically, we evaluated the impact of pre-processing on the quality and quantity of extracted DNA, PCR amplicon, resulting HTS-reads, microbial diversity, and community composition with the non-processed sediment as the basis of comparison. Given the assumption that membrane filters of different size fractions (i.e., samples filtered from membranes of different pore sizes) retain different microbial biomass, we also assessed the difference in relative abundances, composition, and diversity of microbial taxa retained between each filter fractions.

## Results

### DNA yield, PCR amplicon, and HTS-read abundance

**Fig. 1:**
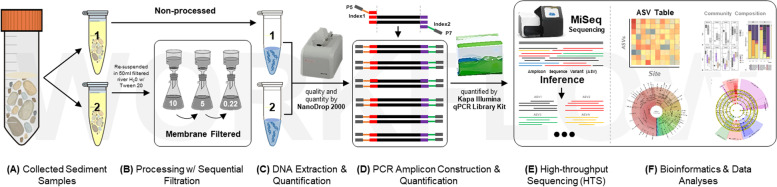
Schematic overview of the experimental procedure of the sediment-associated microbial community profiling employed in this study. **A** Collection of sediment samples. **B** Sequential membrane filtration from 10, 5 to 0.22 μm pore size filters as pre-processing step. **C** DNA extraction following the protocol of Zhou et al. (1996) (as employed in Solomon et al., 2016) with some modifications. **D** One-step PCR amplification of the 16S rRNA V4 hypervariable region. **E** Sequencing through the Illumina MiSeq Platform. **F** Bioinformatics and statistical data analysis were done in R (R Core Team, 2019)

The sediment samples assessed in this study were collected from three sites (i.e., sites A and C are from up-welling zones; site B from a down-welling zone) on selected gravel bars in the Trinity River assessed in the study of Serrana et al. [38]. The experimental procedure of the sediment-associated microbial community profiling employed in this study is illustrated in Fig. 1. Non-processed sediment samples (also indicated as NP) are the reference group and serves as the baseline comparison of the pre-processed sediment samples that underwent sequential membrane filtration from a pre-filter (10 μm pore size), mid-filter (5 μm) and a final collection filter (0.22 μm).

The initial concentration and absorbance ratio (at 260/280 and 260/230) to assess extracted DNA purity [39] were measured via spectrophotometry (Table 1; Figure S1a and b). The DNA yield between sites (A, B, and C) and filters (NP, 10, 5, and 0.22) was higher for sites A and B, and NP and 0.22 filters, but a significant difference between the observed values were only reported for the sites. A ratio of ~ 1.8 is generally accepted as pure DNA for the 260/280 ratio. Although sites B and C and filters 10 and 5 reported a relatively high 260/280 ratio, ANOVA showed no significant difference in DNA purity between sites and between filters. The 260/230 ratio was also relatively low for all samples, given the accepted range of 2.0–2.2 for pure nucleic acid indicative of the presence of contaminants, e.g., EDTA, carbohydrates, and phenol. It was notable that the mean PCR amplicon library concentration of NP was relatively lower than those of the filtered samples, given that it has a higher extracted DNA concentration. However, the PCR amplicon library concentrations quantified via qPCR were not significantly different between sites and between filters. The correlation between extracted DNA and PCR amplicon library concentrations was not significant (Pearson correlation: *r* = -0.024, *p* = 0.94) (Figure S2).

**Table 1:** Quality and quantity of extracted DNA, PCR amplicon, and HTS-read and amplicon sequence variant (ASV) count per sediment sample

Site	Code	Filter Type	Extracted DNA^a^			Amplicon Library			Read Processing				Taxonomic Assignment	
						**qPCR (nM)**^**b**^	**DNA Assay**^**c**^							
			**ng/ul**	**A**_**260**_**/A**_**280**_	**A**_**260**_**/A**_**230**_		**Size (bp)**	**Band**	**Raw Reads**	**Quality Filtered**	**Denoised**	**Non-Chimeric**	**Reads w/ Tax. ID**	**ASVs w/ Tax ID**
A	ANP	NP	307.1	1.40	0.46	4.88	410	1	1,166,991	560,891	456,284	446,586	424,397	461
	A02	0.22	479.7	1.37	0.61	14.16	409	2	305,045	171,106	154,579	147,490	143,019	436
	A05	5	110.1	1.43	0.50	45.97	408	1	73,962	40,433	32,181	31,458	30,146	183
	A10	10	619.2	1.45	0.37	56.53	413	1	260,341	148,596	112,796	105,220	96,643	633
B	BNP	NP	625.5	1.45	0.30	56.79	413	1	446,984	135,890	87,857	86,948	85,109	75
	B02	0.22	397.9	1.61	0.75	27.33	414	1	79,771	29,699	22,339	22,153	20,745	104
	B05	5	44.3	2.53	0.06	8.34	415	1	146,808	101,535	86,288	82,169	77,590	465
	B10	10	35.8	3.08	0.04	17.38	415	1	182,665	135,387	125,620	112,804	105,066	1,071
C	CNP	NP	107.1	1.67	0.13	1.44	396	1	790,386	381,687	285,719	275,483	113,715	426
	C02	0.22	2.3	1.86	0.09	76.9	412	2	112,123	61,001	54,375	50,476	46,631	295
	C05	5	5.1	1.03	0.17	43.23	411	3	23,323	14,327	12,515	10,757	9,582	141
	C10	10	7.6	5.10	0.03	4.40	388	2	217,176	138,449	97,116	94,199	89,877	460
**Total**									**3,805,575**	**1,919,001**	**1,527,669**	**1,465,743**	**1,242,520**	**2,875**

^a^Initial quantification and quality assessment of extracted DNA via NanoDrop Spectrophotometer

^b^Amplicon library quantification via Kappa Illumina Library Quantification Kit

^c^DNA Assay for fragment size quantification and quality via Agilent 2100 BioAnalyzer High Sensitivity DNA Kit. "NP" stands for non-processed sediment samples; "10" for the pre-filter (10 μm), "5" for the mid-filter (5 μm), and "0.22" for the collection filter (0.22 μm)

Based on the site and filter grouping, sites A and C and filters NP, 10, and 0.22 had higher read abundances (from raw reads to reads with taxonomic assignment) and ASV counts than site B and filter 5, respectively (Figure S3 and Table S1). ANOVA showed no significant difference in read and ASV counts between the sites, while the raw, filtered (ANOVA; *p* < 0.05), denoised, and non-chimeric reads (ANOVA; *p* < 0.10) were significantly different between the filters. Although the amplicon libraries were normalized to equimolar concentrations before HTS, the NP samples had significantly higher absolute raw read abundance than the filtered samples (t-test: *p* < 0.05). After quality filtering, NP was only significantly different from filter 5 (t-test: *p* = 0.047). Furthermore, the correlations between the read abundances from raw reads to each processing step were all significantly (*p* < 0.05) positive with strong (Pearson's *r* > 0.60) to very strong (Pearson's *r* > 0.80) correlations (Figure S2).

### ASV richness, taxonomic diversity, and community composition

**Fig. 2:**
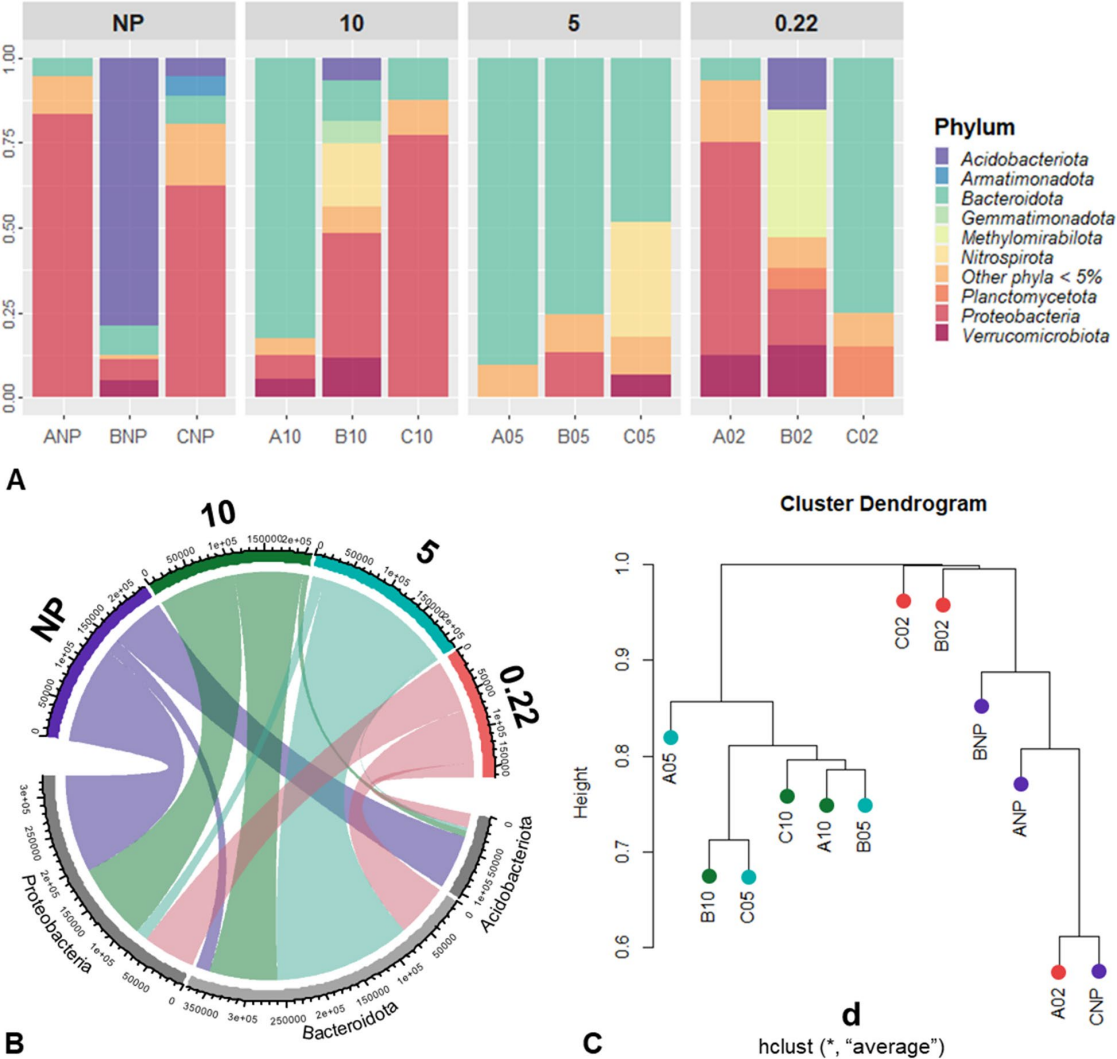
Microbial community composition. **A** Relative abundance of microorganisms identified by 16S rRNA amplicon sequencing. Compositions are illustrated at the phylum level. **B** The chord diagram indicating the log-transformed abundance of the top three Phylum detected for each filters. **C** Hierarchical clustering dendrogram of the similarity in community composition across the sampling sites. Color codes: blue for the non-processed (NP) sediments; green for the pre-filter (10 μm); teal for the mid-filter (5 μm); and red for the collection filter (0.22 μm)

From the 2,875 ASVs, 2,871 were identified as bacteria, while 4 ASVs were assigned as archaea (i.e., Nitrosopumilales and Woesearchaeales) for all sampling sites. We identified a total of 324 microbial genera from 232 families under 161 orders, 85 classes, and 39 phyla, including unclassified taxa (e.g., Unclassified Bacteria). Figure 2A presents the relative abundance of the sediment-associated microbial phyla grouped per filter. Phyla with high relative sequence abundances include Proteobacteria, Bacteroidota, and Acidobacteria (Fig. 2B). Rhodobacteriaceae and Vicinamibacteriaceae predominantly represented non-processed sediments. Whereas Chitinophagaceae, Microscillaceae, and *Flavobacterium* dominate the 10, 5, and 0.22 filters, respectively (Figure S4).

**Fig. 3:**
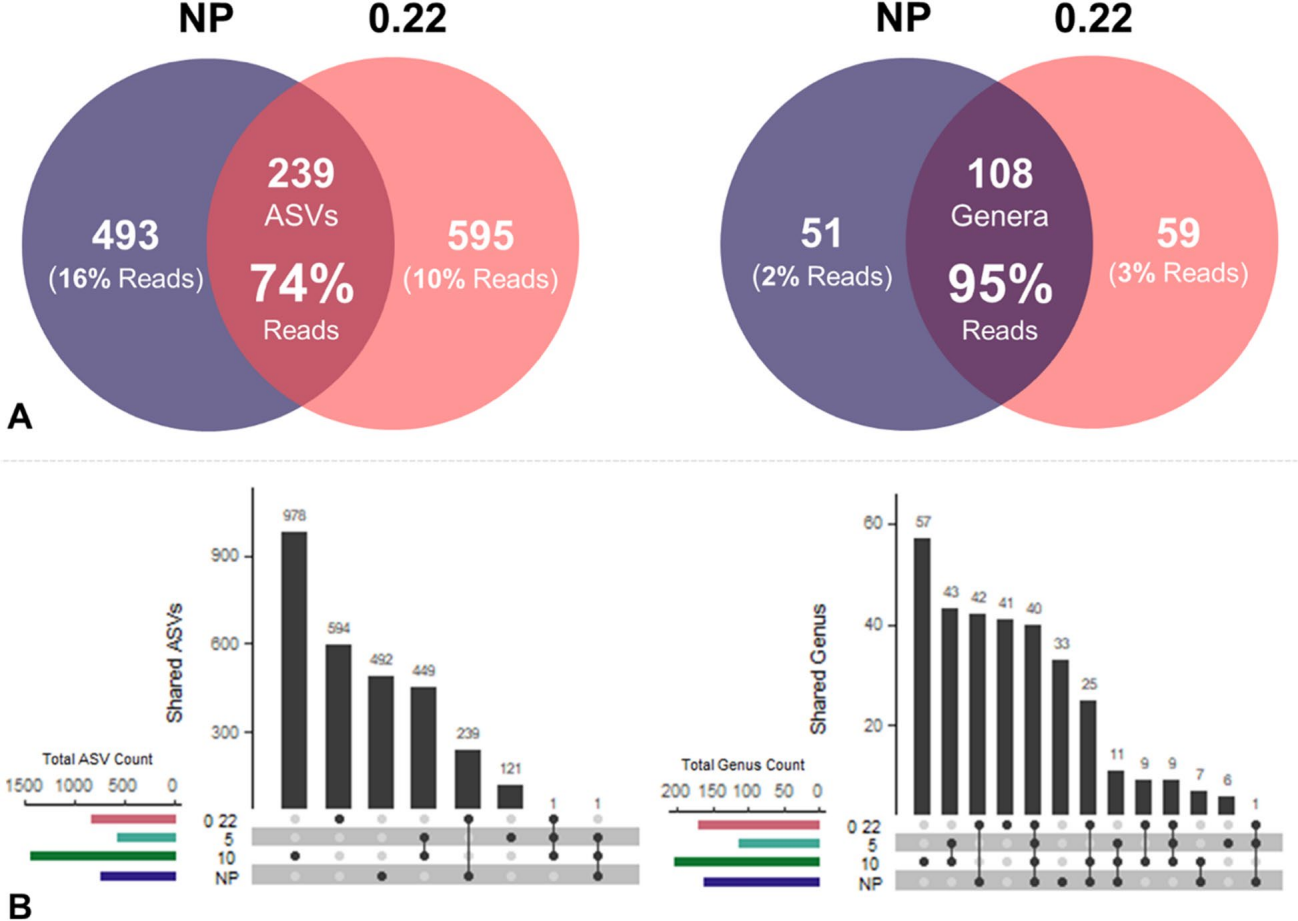
Shared and unique ASVs and genus presented in (**A**) venn diagrams and (**B**) UpSetR plots between the non-processed (NP) and pre-processed samples (represented by the collection filter, 0.22 μm), and between all groups (NP, 10, 5, and 0.22 μm) of sediment samples. Each column corresponds to number of ASV/genera that are present in each group denoted by the dark circles

To explore the difference between the non-processed and collection filter samples, the shared and unique ASVs and taxa (e.g., Phylum, Class, Order, Family, and Genus) assigned per filter were visualized via Venn diagrams (Fig. 3A and Figure S5) and UpSetR plots (Fig. 3B and Figure S6). Notably, the 10 filters always showed the highest ASV count throughout the sites (Table 1). When grouped by filter type, the 10 filters had the highest unique ASV count with 978, followed by 0.22, NP, and 5 with 594, 492, and 121 unique ASVs, respectively. The NP and 0.22 collection filters shared 63 ± 89 (Mean ± SD) or a total of 239 ASVs (74% of reads shared) having 257 ± 143 (total of 493; 16% of reads) and 215 ± 81 (total of 595; 10% of reads) unique ASVs, respectively. When aggregated at the genus level, the two methods shared 35 ± 34 or a total of 108 genera (95% of reads) with 54 ± 40 (total of 51; 2% of reads) and 39 ± 1 (total of 59; 3% of reads) unique genera, respectively. With NP as reference, ASV or taxa that are not detected from the 0.22 filter are referred to as false-negative, while those that are only present from the latter are referred to as false-positive detections. Also, the 10 and 5 filters shared 449 ASVs, and no ASV was shared between all four filters.

Alpha diversity based on Chao1 richness, Shannon diversity, Pielou's evenness, Berger-Parker’s dominance, and the rarity index are presented in Figure S7. ANOVA showed no significant difference between the sites and between filters in richness, diversity, evenness, dominance, and rarity estimates. The NMDS ordinations of the genus and ASV datasets indicated that the samples cluster based on the filters as visualized in the ordination space (Figure S8). Notably, filters 10 and 5, and NP and 0.22 clustered closely together. The hierarchical clustering of samples based on the ASV dataset also showed the separation of NP and 0.22 against the 10 and 5 filters (Fig. 2C). However, PERMANOVA showed no significant difference in the community composition of both the genus (*R*^*2*^ = 0.21, *p* = 0.245) and ASV (*R*^*2*^ = 0.22, *p* = 0.062) datasets.

### Indicator taxa analysis

**Fig. 4:**
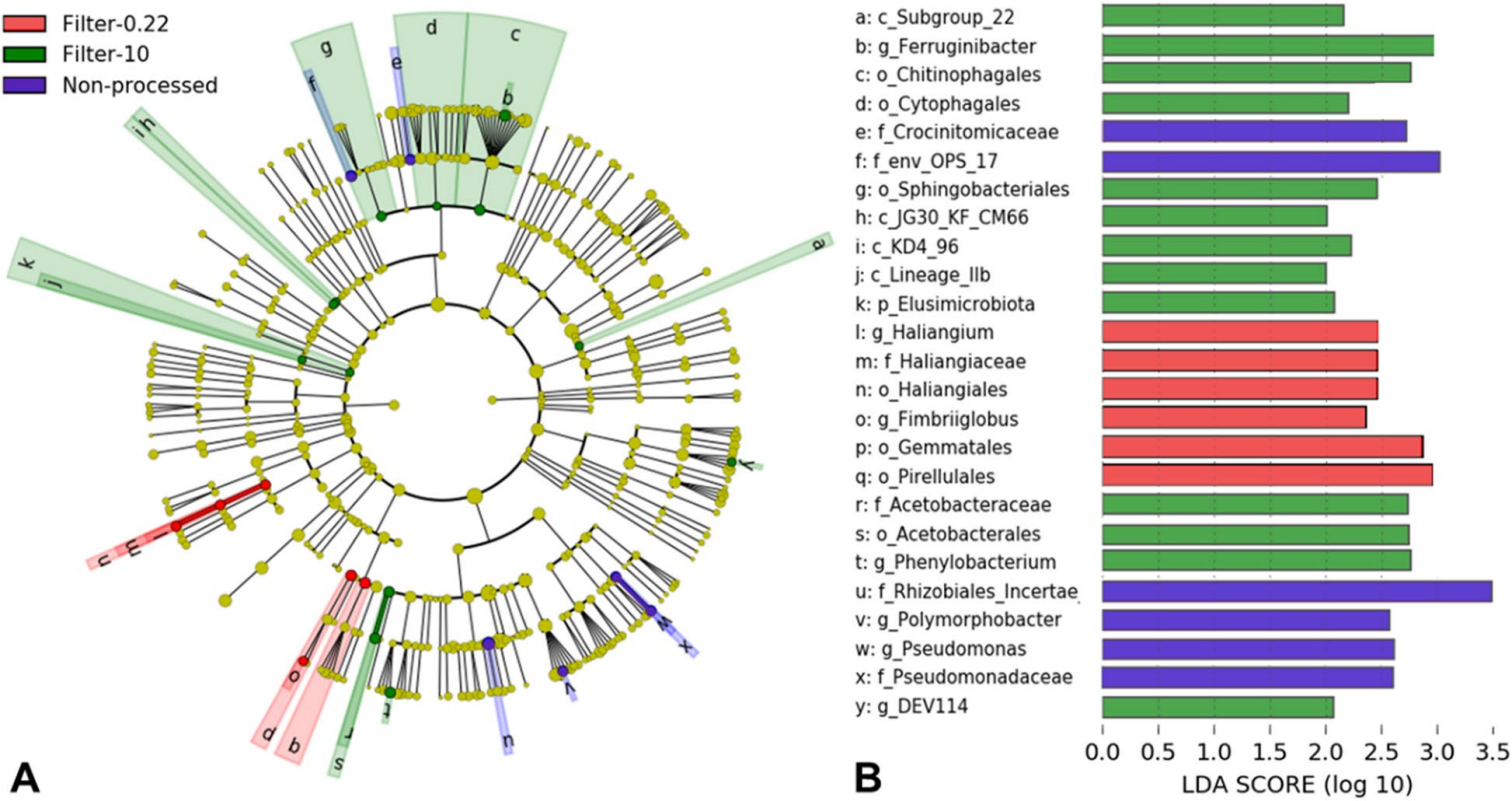
Linear Discriminant Analysis (LDA) Effect Size (LEfSe) plot of indicator taxa identified from non-processed (NP), and sequential filtered (10, 5, and 0.22 μm) sediment samples. **A** Cladogram representing the hierarchical structure of the indicator taxa identified between the non-processed and filtered samples (filter). Each filled circle represents one indicator taxa. Blue, indicator taxa statistically overrepresented in "NP"; red indicator taxa statistically overrepresented in "0.22"; green, indicator taxa statistically overrepresented in "10". **B** Identified indicator taxa grouped by filter and ranked by effect size. The threshold for LDA score was > 2.0. The letter before the taxa indicates taxonomic level: “p_” for phylum; “c_” for class; “o_” for order; “f_” for family; and “g_” for genus

Linear discriminant analysis of effect size (LEfSe) was performed to identify the taxa that significantly explained differences in community composition between the groups. Thirty-five significantly discriminative features out of 51 were selected before internal Wilcoxon, and 25 had an LDA score > 2. A cladogram showing the 25 microbial taxa's phylogenetic distribution significantly associated with each filter group is presented in Fig. 4A. The corresponding linear corresponding analysis (LDA) values for each taxon are shown in Fig. 4B. LEfSe analysis showed that the taxa from four families (i.e., Crocinitomicaceae, Env. OPS 17, Pseudomonadaceae, Rhizobiales *Incertae Sedis*), and two genera (i.e., *Polymorphobacter*, *Pseudomonas*) were significantly abundant in NP compared to other filter groups. For the sequential membrane filters, phylum Elusimicrobiota, four classes [e.g., Subgroup 22 (Acidobacteriota), JG30-KF-CM66 (Chloroflexi)], four orders (e.g., Chitinophagales, Sphingobacteriales), family Acetobacteraceae, and three genera [i.e., DEV114 (Pedosphaeracea), *Ferruginibacter*, *Phenylobacterium*] were significantly more abundant for the 10 μm filter, while three orders (i.e., Gemmatales, Haliangiales, Pirellulales), family Haliangiaceae), and two genera (i.e., *Haliangium*, *Fimbriiglobus*) were significantly more abundant for the 0.22 μm filter. No taxa were found to be significantly abundant for the 5 μm filter.

## Discussion

This study assessed whether freshwater sediment-associated microorganisms would differ between non-processed and pre-processed samples by sequential membrane filtration. We provided the first comparison of the two approaches using 16S rRNA amplicon sequencing for microbial community profiling.

### Influence on the quality and quantity of extracted DNA, PCR amplicon and HTS-reads

The isolation and capture of good quality and quantity DNA from sediment samples are very challenging [9, 10], and the preservation medium and the time between collection and storage is critical for particle or sediment-associated microorganisms to prevent biased overgrowth and DNA damage before HTS sample processing [36]. We observed that extracted DNA concentration varied between sites and filters and was relatively high for the non-processed samples, with the sites having significant difference in DNA yield and no significant difference observed between filters. The difference in DNA yield could be attributed to the difference of the ecological conditions between the gravel bar sites. PCR amplicon concentration and quality were also not significantly different between the non-processed and processed samples. We should note that we used the same DNA extraction method for both non-processed and processed samples, employing the method of Zhou et al. [40], which includes the removal of PCR inhibitors, i.e., humic compounds. The chosen DNA extraction method could present different impacts on the characterization of the overall microbial community composition [41]. Previous studies have investigated the influence of filter types and pore sizes on DNA yield from aquatic ecosystems, i.e., environmental DNA [e.g.,^42^,^43^]. Filters of different pore sizes did not affect the amount of total DNA recovered and detected species from environmental DNA [43].

The PCR amplicon libraries were normalized before sequencing to ensure an even read distribution for all samples. However, the raw HTS-reads and quality-filtered reads were significantly different between methods, with the non-processed significantly having the highest raw read abundance. Interestingly, after the denoising and the chimeric-read filtering steps, the retained reads from the non-processed sample declined and were not significantly different between methods. This suggested that the retained read abundance after the bioinformatics step was not significantly influenced by sediment processing or lack thereof. Previous studies have reported that higher GC content and longer fragment length decreased the abundance of reads retained after quality filtering [44]. Moreover, fragment length may also impact the base qualities of Illumina reads [45]. The decline in read abundance of non-processed samples (from being significantly different from the others to insignificant difference) after quality filtering suggests the possibility of the extracted DNA having either high GC content or longer fragments, which reduced the reads' base qualities.

### Shared and unique ASVs and taxa between methods and filter fractions

We report that although the non- and pre-processed samples (represented by the final collection filter, 0.22) had more unique than shared ASVs, the latter accounted for 239 ASVs that includes 74% of the reads between the two methods. More so, at the genus level, the non- and pre-processed samples had a relatively high percentage of total shared genus count (108 genera, 50%) that accounts for 95% of the reads' absolute abundance. This showed that the final collection filter (0.22) captured most of the abundant genera identified from the non-processed samples. Notably, the collection filter detected a total of 59 more unique genera (3% of the reads). These false-positive detections suggested that the pre-processed samples can detect taxa not captured from the non-processed approach.

A range of mechanisms potentially drove this false-positive detection. First, this could be due to the effectiveness of the multiple filtration process to reduce inhibitory compounds. Sequential-filter isolation techniques have been employed to improve the yield of environmental DNA by reducing the concentration of inhibitory compounds, e.g., humic acid, polysaccharides, metals, etc. [10, 17, 46]. Specifically, sediment samples contain high humic substances, which are the primary compounds co-extracted with DNA that inhibits enzymes (e.g., *Taq* polymerase) in PCR reactions [47]. The reduction of these inhibition compounds could have led to false-positive taxa in relation to the non-processed samples. However, we observed no significant difference in the quality of extracted DNA to support reduced inhibitory compounds' influence on the false-positive detections.

Other reasons, e.g., sequencing depth (the total number of usable reads from the sequencing machine), have been reported to influence the rate of false-positive detections in metabarcoding studies [48]. Insufficient sequence depth could also result in undetected rare taxa. For example, singletons (single sequence detection or an OTU/ASV only present in one sample) are usually considered erroneous sequences or artefacts and are usually removed for subsequent analysis. The pre-processing might more effectively filter biomass of abundant taxa because of their high aggregation in the environment, resulting in increases of relative reads’ abundances of rare taxa in the samples and false-positive detections. Also, method-specific or unique taxa could result from having abundant taxa with polymorphisms or rare variants [49]. On the other hand, setting a more stringent parameter for quality filtering could reduce the rate of detecting false positives [50, 51]. Given that we employed a relatively lax read quality filtering parameter in this study, the false positive detection could result from low-quality passing reads.

The false-negative taxa (51 genera; 2% of the reads) absent from the collection filter could be microbial groups that passed through the 0.22 μm pore-sized filter. As previously reported by Maejima et al. [52], isolated bacteria from lake water samples belonging to the Proteobacteria, Bacteroidetes, Firmicutes, and Actinobacteria can be small enough to pass through a 0.22 μm pore size filter. The filtered fractions from < 0.2 μm filtered samples that were usually considered “sterile” were found to still contain miniature cells, ultramicrobacteria (i.e., bacteria whose cell size are smaller than 0.1 μm^3^) and slender filamentous bacteria (e.g., Oligoflexia, Proteobacteria) overlooking a broad diversity of filterable agents [53, 54]. However, we observed that the false-negative taxa had very low read abundance, which could be due to smaller cell size leading to low DNA yield. This suggests that the microbial groups that possibly passed through the 0.22 μm pore-sized collection filter were mostly low abundant taxa. These additional detections may prove helpful when assessing rare taxa from the sediment samples. Nonetheless, we observed a low read abundance of these false-positive and negative detections proving that the most abundant taxa of the sediment samples are detected on both methods. As demonstrated from the diversity and community composition analyses employed in this study, these method-specific taxa would unlikely affect these results.

On the other hand, the pre- and mid-filters had a relatively high count of 449 shared and 978 and 121 unique ASVs, respectively. The non-processed samples only had 1 ASV shared with the pre- and mid-filter, similar to the collection filter. The clear separation between NP and 0.22 against the 10 and 5 filters was also observed in the NMDS ordination and the hierarchical clustering. At the genus level, the pre- and mid-filters had 57 and 6 unique genera. These values added with the genera shared between the two filters make a total of 106 captured solely from the pre- and mid-inline filtration. The very low ASV and low genera shared between non-processed and collection filters against the pre- and mid- filters suggested that a huge part of the sediment microbial community is underrepresented or lost from the community profile during the pre-processing. A previous study comparing the prokaryotic and eukaryotic diversity and community composition between pre- and collection filters from lake water samples suggested the possible “pre-filter” bias in the community structure from the collected biomass [55]. They reported contrasting read abundance even though most operational taxonomic units (OTUs) were shared between filters. Sequential filtration of sediments might be a stochastic process where taxa are presumably retained according to cell size rather than their abundance, with the rare taxa retained along the previous filtration step [56]. We presented a stronger pre- and mid-filter community composition bias, given that very few ASVs and taxa were shared between the in-line filters and the non- and pre-processed samples. Since we observed that certain sediment-associated microbial taxa were not captured from the non-processed samples, and if only the collection filter is considered to represent the pre-processed samples' microbial community profile, we suggest the inclusion of pre-filters in microbial communities' profiling.

### Microbial community and taxonomic difference between methods and filter fractions

Statistical analyses revealed that groups based on filter were not significantly different in the richness, diversity, and evenness estimates of alpha diversity. Although shared taxa between the two methods were relatively low, community structures based on Bray–Curtis distance were also not significantly different between the two methods. Bray–Curtis dissimilarity is sensitive to differences in abundance between taxa, where abundant taxa are weighted more than the rare ones [57]. Although the overall microbial community composition was not significantly different between the two methods, the significantly abundant indicator taxa detected between the filter types were different, primarily due to the variations in detecting low abundance taxa.

Based on LEfSe, representatives from the Alphaproteobacteria (i.e., Rhizobiales *Incertae Sedis* and *Polymorphobacter*), *Pseudomonas* (Pseudomonadaceae), and the Crocinitomicaceae and the uncultured eubacterium env. OPS 17 were significantly more abundant in the non-processed sediment samples. The taxa affiliated with the Alphaproteobacteria have shown a consistent preference for a particle-attached lifestyle [58]. The pre-filter (10 μm filter) had significantly more abundant taxa with representatives from Acetobacteraceae (Alphaproteobacteria), Acidobacteriota, Bacteroidota, Chloroflexi, and Elusimicrobiota. Candidate microbial divisions and Chloroflexi have been reported to be primarily recovered when particle samples were subjected to filtration in situ [35]. The collection filter (0.22 μm filter) had significantly more abundant *Fimbriiglobus* (Gemmatales), Pirellulales, and *Haliangium* (Haliangiales) sequences. The first two taxa are classified as members of the Planctomycetes, while the latter belong to the Myxococcota. A study evaluating the influence of standard filtration practices on marine particles also reported that proportional abundances in the pre-filter fraction of Myxococcales (Deltaproteobacteria) and Planctomycetes increased with filter volume [20]. Furthermore, in-situ filtration (0.4 μm filter) increased the capture of Planctomycetes by fivefold compared to on-ship in-line filtration [35].

## Summary and conclusion

We found no significant difference in the quantity and quality of extracted DNA and PCR amplicon between non- and pre-processed sediment samples in the present study. Raw and quality-filtered reads were significantly different between methods, but read abundance after bioinformatics processing was not significantly different. These results suggest that read abundance after the bioinformatics steps was not significantly influenced by sediment processing or lack thereof. We report that although the non- and pre-processed sediment samples had more unique than shared ASVs, both methods shared a total of 239 ASVs that accounts for 74% of the reads. More so at the genus level, the final collection filter also detected most of the genus identified from the non-processed samples, with 51 false-negatives (2% of the reads) and 59 false-positive genera (3% of the reads). The alpha diversity indices estimated, and the microbial community composition was not significantly different between the non- and pre-processed samples. These results demonstrate that while differences in shared and unique ASVs and microbial taxa were detected, both methods revealed comparable microbial diversity and community composition. We also suggest the inclusion of sequential filters (i.e., pre- and mid-filters) in the community profiling, given the additional taxa not detected from the non-processed and the final collection filter. We presented the feasibility of pre-processing sediment samples for community analysis and the need for further assessment sampling strategies to help conceptualize appropriate study designs for sediment-associated microbial community profiling.

Our time from collection to processing and ethanol preservation of the filtered samples was from two to four hours. Previous studies reported that a larger processing time between sample collection and filter storage might allow the growth of opportunistic prokaryotic groups introducing bias by microbial population turnover within the sample. Here, the sediments processed for sequential membrane filtration were from samples that have already been preserved in ethanol; hence, this bias was not tested in our experimental design. In addition, it is still worth noting that the difference in detection of certain taxa between groups could have been influenced by the difference in sampling site since the sediment samples used in this study were collected from different gravel bars. However, we did not assess sediment characteristics, e.g., particle size and organic matter concentration, so we cannot fully infer that the observed sampling site differences was affected by sample type. Hence, our results observed from freshwater sediments may be different for sediments or particle-associated microorganisms collected from other systems, given that the magnitude and exact mechanism of sample type biases may likely be influenced by various factors, e.g., particle load, bulk microbial abundance, etc. We recommend further assessment of sediment pre-processing by comparing different filter types and combinations, preservation medium, sample volume, sediment type and the influence of various processing times for further method evaluation. This will fully present the capability and viability of on-site sequential membrane filtration as a processing step against the direct collection and preservation of freshwater sediment samples.

## Methods

### Sediment collection and sample pre-processing

Sediment samples from three sites (i.e., sites A and C are from up-welling zones; site B from a down-welling zone) were collected approximately 10 cm below the submerged surface of selected gravel bars in the Trinity River assessed in the study of Serrana et al. [38]. The Trinity River is a large gravel-bed river impounded by the Trinity Dam (164 m a.b.l. and 3020 million m^3^ storage) and the smaller Lewiston Dam (28 m a.b.l. and 18 million m^3^ storage) in northern California, USA. It is under current dam operating guidelines with a mean annual flood of approximately 180 m^3^/s [59]. The experimental procedure of the sediment-associated microbial community profiling employed in this study is illustrated in Fig. 1.

The collected sediment samples were mainly composed of coarse sediments ranging from 1 to 5 mm in diameter, containing smaller sand grains and fine particulate mass. The samples were stored in 50 ml sterile falcon tubes and immediately fixed with 99.5% molecular grade ethanol upon collection. Pre-processing of sediment samples was done two to four hours after collection. Subsamples of ~ 600 mg were aliquoted for sequential membrane filtration. The subsamples were resuspended in separate 50 ml solutions containing 0.22 μm filtered river water with Tween 20 (at a concentration of 1 ml l^−1^ v/v), agitated and mixed via a magnetic stirrer for 30 min. The resuspended subsamples were then filtered through a pre-filter with a 10 μm pore size (Nuclepore™ hydrophilic membrane filter paper; Whatman, Tokyo, Japan), followed by a mid-filter of 5 μm pore size (Mixed cellulose ester membrane filter; Merck Millipore, USA) and finally through a 0.22 μm collection filter (Cellulose mixed ester membrane filter; Merck Millipore, USA). The pre-processed samples were then kept in 2 ml microcentrifuge tubes, immediately fixed with 99.5% molecular grade ethanol. For non-processed sediments, triplicate subsamples of 200 mg were taken from the collected samples preserved in 50 ml Falcon tubes with 99.5% molecular grade ethanol.

### DNA extraction, PCR amplification, and sequencing

Before DNA extraction, the membrane filters were removed from the collection tubes and dried at room temperature until most of the preserving ethanol evaporated. The membrane filter tubes (ethanol with finer particulate mass) and the subsampled non-processed sediments were then subjected to high speed (12,000 rpm) centrifugation for 30 min to resuspend the remaining fine particles and sediments to the bottom of each tube. The supernatant was removed carefully, and the tubes were dried at room temperature to evaporate the remaining ethanol. The dried membrane filters were cut into smaller pieces using sterile scissors and placed back into their original tubes. The samples were then suspended in a buffer consisting of 10 mM EDTA, 50 mM Tris–HCl, 50 mM Na_2_HPO4·7H_2_O at pH 8.0 to remove PCR inhibitors [40, 60]. Genomic DNA was extracted from both the non-processed and filtered subsamples following Zhou et al. [40] as employed in Solomon et al. [10]. The extracted DNA of the subsamples were pooled and quantified using a NanoDrop spectrophotometer (NanoDrop 2000, Thermo Scientific). This served as the template for subsequent library preparation and amplicon sequencing.

Amplicon library preparation was carried out through a one-step PCR amplification using modified fusion primers of the V4 hypervariable region of the 16S SSU rRNA gene, i.e., 515F and 806R [61]. The PCR was performed with high-fidelity Phusion polymerase (Thermo Fisher Scientific Inc.) in a T100 Thermal Cycler (Bio‐Rad Laboratories, USA). The 25 μl PCR reaction mixture consisted of five μl of 5X Phusion GC Buffer, 1.25 μl each of the forward and reverse primers (10 μM), two μl dNTPs (2.5 mM), 0.75 μl DMSO, 0.25 μl Phusion Polymerase (1 U) and one μl of template DNA. The PCR condition followed was initial denaturation at 98 °C for 3 min, 25 cycles of denaturation at 98 °C for 15 s, annealing at 50 °C for 30 s, and extension at 72 °C for 30 s, followed by a final extension period at 72 °C for 7 min.

Post-amplification, library-quality control was performed by checking the library size distribution via the High-Sensitivity DNA chip (Agilent BioAnalyzer). The libraries were purified and size selected using SPRI beads (AmpureXP, Beckman Coulter Genomics). Amplicon size was ~ 400-bp. Triplicate quantitative PCR reactions at appropriate dilutions were performed to quantify the amplicon libraries with the KAPPA Illumina Library qPCR Quantification kit (Kappa Biosystems, Wilmington, MA, USA). Negative control was used to monitor contamination from DNA extraction and PCR to post-amplification library quantity and quality verification; however, no quantifiable amplicon was detected for further analysis. The purified amplicon libraries were then normalized, and equimolar amounts were pooled. The 4 nM pooled library was sequenced at the Advanced Research Support Center (ADRES) of Ehime University using the Illumina MiSeq platform with paired-end reads of 300-bp per read.

### Read processing and taxonomic assignment

The raw sequence reads generated on the Illumina MiSeq platform were demultiplexed via the command-line tool Cutadapt v.2.1 [62]. The 3,805,575 demultiplexed sequences were quality screened, processed, and inferred amplicon sequence variants (ASVs) with the denoising pipeline of the DADA2 v.1.12 package [63] in R v.3.6.2 [64]. Based on the read error profiles, the reverse reads have poor read quality. Low read abundance with acceptable overlaps between the reads can be accounted for after quality filtering; therefore, only the forward reads were used in the subsequent analysis. Primer contaminants were excluded, and the reads were filtered based on quality and identified sequence variants likely to be derived from sequencing error. ASVs were inferred from the sequence data, subsequently removing chimeric sequences and singletons. The DADA2 pipeline was implemented to use sequence error models to correct amplicon errors in ASVs. Reads with a maximum expected error greater than 5 were discarded as a quality filtering measure and truncated at a read length of 100-bp. The remaining ASV sequences were aligned to the SILVA 138 database [65] through the SILVA ACT: Alignment, Classification, and Tree Service online server (www.arb-silva.de/aligner) [66]. For this analysis, the small subunit (SSU) category was selected, and a minimum similarity identity of 0.95 was set with ten neighbors per query sequence. Sequences below 70% identity were rejected and discarded. The least common ancestor (LCA) method was used for the taxonomic assignment. Chloroplasts, mitochondria, and unclassified ASVs were removed, resulting in a total of 2,875 taxonomically assigned ASVs.

The raw sequence data were deposited into the National Center for Biotechnology Information (NCBI) Sequence Read Archive (SRA) under the accession number PRJNA559761. The ASV matrix, the taxonomy, and the sample table generated in this study have been deposited in the Figshare data repository (https://doi.org/10.6084/m9.figshare.13088834) [67].

### Statistical analysis and data visualization

Statistical analyses were performed using various packages available in R v.3.6.2 [64]. The significant differences in the quality and quantity of extracted DNA and PCR amplicon libraries, and the HTS-reads for each read processing steps between sites (i.e., A, B, C), and filters [i.e., non-processed (NP), pre-filter (10 μm filter, "10"), mid-filter (5 μm, "5"), and the collection filter (0.22 μm, "0.22")] were tested via two-way analysis of variance (ANOVA), and pairwise comparisons via multiple T-tests in the presence of significant main effects using the stat_compare_mean() in the ggpubr package [68]. The correlation between the extracted DNA and PCR amplicon library concentration and purity and between HTS-read count per processing step (i.e., raw reads, quality filtering, denoising, chimera removal, taxonomic assignment, and ASV count) were tested with Pearson correlation analyses on log-transformed data. A correlogram with significant tests was calculated and visualized with the Hmisc and corrplot packages [69].

Before subsequent statistical analyses, the ASV table was normalized at median sequencing depth. The shared and unique taxonomic assignment and ASVs between the groups were visualized with Venn diagrams and UpSetR plots [70]. The boxplots were illustrated via ggplot2 [71]. The spatial differences between the microbial communities were visualized using non-metric dimensional scaling (NMDS) based on Bray–Curtis distances with the plot_ordination() function from the phyloseq package [72], and in a hierarchical clustering dendrogram based on the average-linkage algorithm using the hclust() function. PERMANOVA (permutational multivariate analysis of variance) [vegan; 73] was performed to identify significant differences in community composition between filters based on the NMDS ordination.

Alpha diversity metrics (i.e., Chao1 richness, Shannon diversity, Pielou's J evenness, Berger-Parker’s dominance, and rarity index) were calculated and visualized based on the ASV dataset to identify the changes in community structure between the non-processed and filtered samples using the plot_alpha_diversities() function [microbiomeutilities; 74Significant differences between the alpha diversity of sites and filters were also tested via ANOVA and pairwise comparisons via multiple t-tests in the presence of significant main effects. Linear discriminant analysis (LDA) effect size (LEfSe) was performed using the python’s LEfSe package [75] (parameters: *p* < 0.05, q < 0.05, LDA > 2.0) to identify which microbial taxa significantly explained differences in community composition between the filter groups (i.e., NP, 10, 5, 0.22). The LEfSe algorithm was used to determine indicator taxa considering both the abundance and occurrence of a particular taxon. Identifying differentially abundant taxa using LEfSe analysis is specifically designed for categorical group comparisons of microbiome data, and will provide additional support on the effects of pre-processing compared to the non-processed sediment samples on the detection of significantly abundant taxa.

## Supplementary Information

**Additional file 1: Table S1.** Read and amplicon sequence variant (ASV) abundance grouped by filter [mean average (standard deviation)]. **Figure S1.** Box-and-whisker plots of extracted DNA concentration and quality (log-transformed) grouped by site **(A)** and filter **(B)**. The p-value presented were from the analysis of variance (ANOVA) tests between samples. **Figure S2.** Pearson’s correlation matrix on log-transformed values. Statically significant (*p* < 0.05) Pearson’s R values are highlighted. “conc.” Stands for extracted DNA (ng/μl); “A280” for 260/280 ratio of DNA purity; “A230” for 230/280 ratio of nucleic acid purity; “qPCR” for the PCR amplicon library concentration (nM); “input” for the raw HTS-reads; “filtered” for quality filtered reads; “denoised” for the denoised reads; “nonchim” for the non-chimeric reads; “tax.id” for the reads with taxonomic assignment; “asv.tax” for the ASV count with taxonomic assignment. **Figure S3.** Box-and-whisker plots of log-transformed read and amplicon sequence variant (ASV) abundance grouped by site **(A) **and filter **(B)**. Means with the same letter are not significantly different according to t-test at *p* < 0.05. **Figure S4.** Absolute abundance of the top 10 genera grouped by filter. **Figure S5.** Venn Diagrams showing shared and unique ASVs and genus between the filter types amongst sites. **Figure S6.** UpSetR plots showing shared and unique taxa between the non-processed (NP) and filtered (10, 5, and 0.22 μm) sediment samples. The bars in the upset plot show the overlap between the indicated sample below. **Figure S7.** Box-and-whisker plots of alpha diversity indices metrics comparing the samples by site **(A)**, and filter **(B)**. **Figure S8.** Cluster analysis via non-metric multidimensional scaling (NMDS) based on Bray-Curtis dissimilarity showing microbial community composition the non-processed (NP), and sequential filtered (10, 5, and 0.22 μm) sediment samples for the genus **(A)** and ASV **(B)** datasets.

## Data Availability

The raw sequence data were deposited into the National Center for Biotechnology Information (NCBI) Sequence Read Archive (SRA) under the accession number PRJNA559761. The ASV matrix, the taxonomy and the sample table generated in this study have been deposited in the Figshare data repository (https://doi.org/10.6084/m9.figshare.13088834) [67].

## Abbreviations

ANOVA: Analysis of Variance
ASV: Amplicon Sequence Variants
LDA: Linear Discriminant Analysis
LEfSe: Linear discriminant analysis effect size
NP: Non-processed sediment samples
PERMANOVA: Permutational Multivariate Analysis of Variance
