# Supplementary material for: Sediment-associated microbial community profiling: sample pre-processing through sequential membrane filtration for 16S rRNA amplicon sequencing
Source: BMC Microbiol. 2022 Jan 20;22:33. doi: 10.1186/s12866-022-02441-0 (PMC8772107; doi:10.1186/s12866-022-02441-0)
Supplement: Supplementary file 1 — Additional file 1: Table S1. Read and amplicon sequence variant (ASV) abundance grouped by filter [mean average (standard deviation)]. Figure S1. Box-and-whisker plots of extracted DNA concentration and quality (log-transformed) grouped by site (A) and filter (B). The p-value presented were from the analysis of variance (ANOVA) tests between samples. Figure S2. Pearson’s correlation matrix on log-transformed values. Statically significant (p < 0.05) Pearson’s R values are highlighted. “conc.” Stands for extracted DNA (ng/μl); “A280” for 260/280 ratio of DNA purity; “A230” for 230/280 ratio of nucleic acid purity; “qPCR” for the PCR amplicon library concentration (nM); “input” for the raw HTS-reads; “filtered” for quality filtered reads; “denoised” for the denoised reads; “nonchim” for the non-chimeric reads; “tax.id” for the reads with taxonomic assignment; “asv.tax” for the ASV count with taxonomic assignment. Figure S3. Box-and-whisker plots of log-transformed read and amplicon sequence variant (ASV) abundance grouped by site (A) and filter (B). Means with the same letter are not significantly different according to t-test at p < 0.05. Figure S4. Absolute abundance of the top 10 genera grouped by filter. Figure S5. Venn Diagrams showing shared and unique ASVs and genus between the filter types amongst sites. Figure S6. UpSetR plots showing shared and unique taxa between the non-processed (NP) and filtered (10, 5, and 0.22 μm) sediment samples. The bars in the upset plot show the overlap between the indicated sample below. Figure S7. Box-and-whisker plots of alpha diversity indices metrics comparing the samples by site (A), and filter (B). Figure S8. Cluster analysis via non-metric multidimensional scaling (NMDS) based on Bray-Curtis dissimilarity showing microbial community composition the non-processed (NP), and sequential filtered (10, 5, and 0.22 μm) sediment samples for the genus (A) and ASV (B) datasets. [file 12866_2022_2441_MOESM1_ESM.docx]

# Supplementary Information

Sediment-associated microbial community profiling: sample pre-processing through sequential membrane filtration for 16S rRNA amplicon sequencing

**Authors and Affiliations**

Joeselle M. Serrana^1,2,3^ and Kozo Watanabe^1,2,3*^

^1^Center for Marine Environmental Studies, Ehime University, Bunkyo-cho 3, Matsuyama, Ehime 790-8577, Japan

^2^Graduate School of Science and Engineering, Ehime University, Bunkyo-cho 3, Matsuyama, Ehime 790-8577, Japan

^3^Biological Control Research Unit, Center for Natural Sciences and Environmental Research, De La Salle University, 2401 Taft Avenue, Manila 1004, Philippines

*corresponding author

Prof. Kozo Watanabe, PhD

E-mail Address: watanabe.kozo.mj@ehime-u.ac.jp

Phone & Fax Number: +81 (0) 89 927 9847

### **Table S1.** Read and amplicon sequence variant (ASV) abundance grouped by filter [mean average (standard deviation)].

| **Filter Type** | **Raw Reads** | **Quality Filtered** | **Denoised** | **Non-Chimeric** | **Reads w/**  **Tax. ID** | **ASVs w/**  **Tax ID** |
| --- | --- | --- | --- | --- | --- | --- |
| NP | 801,453 (360,131)^a^ | 359,489 (213,368)^a^ | 276,620 (184,381)^a^ | 269,672 (179,889)^a^ | 207,740 (188,174)^a^ | 320 (213)^a^ |
| 0.22 | 165,646 (121,801)^b^ | 87,268 (74,272)^ab^ | 77,097 (68,986)^a^ | 73,373 (65,730)^a^ | 70,131 (64,435)^a^ | 278 (166)^a^ |
| 5 | 81,364 (62,074)^b^ | 52,098 (44,759)^b^ | 43,661 (38,202)^a^ | 41,461 (36,741)^a^ | 39,106 (34,878)^a^ | 263 (176)^a^ |
| 10 | 220,060 (38,918)^b^ | 140,810 (6,913)^ab^ | 111,844 (14,275)^a^ | 104,074 (9,355)^a^ | 97,195 (7,609)^a^ | 721 (314)^a^ |
| "NP" stands for non-processed sediment samples; "10" for the pre-filter (10 μm), "5" for the mid-filter (5 μm), and "0.22" for the collection filter (0.22 μm). Means with the same letter are not significantly different according to t-test at p < 0.05 (also shown in a boxplot in Supplementary Figure 3B). | | | | | | |


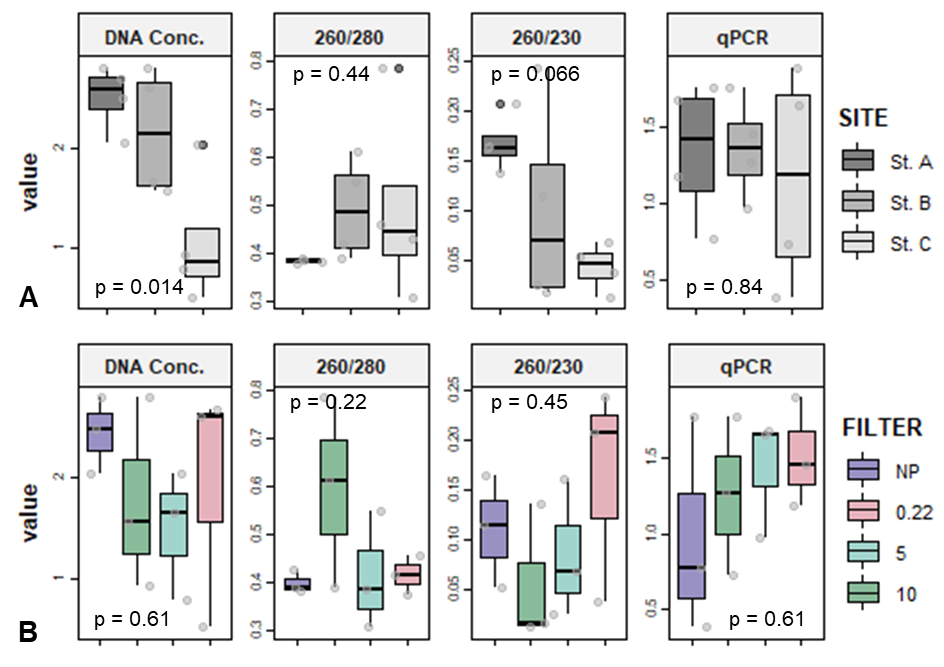


### **Figure S1.** Box-and-whisker plots of extracted DNA concentration and quality (log-transformed) grouped by site **(A)** and filter **(B)**. The p-value presented were from the analysis of variance (ANOVA) tests between samples.

**
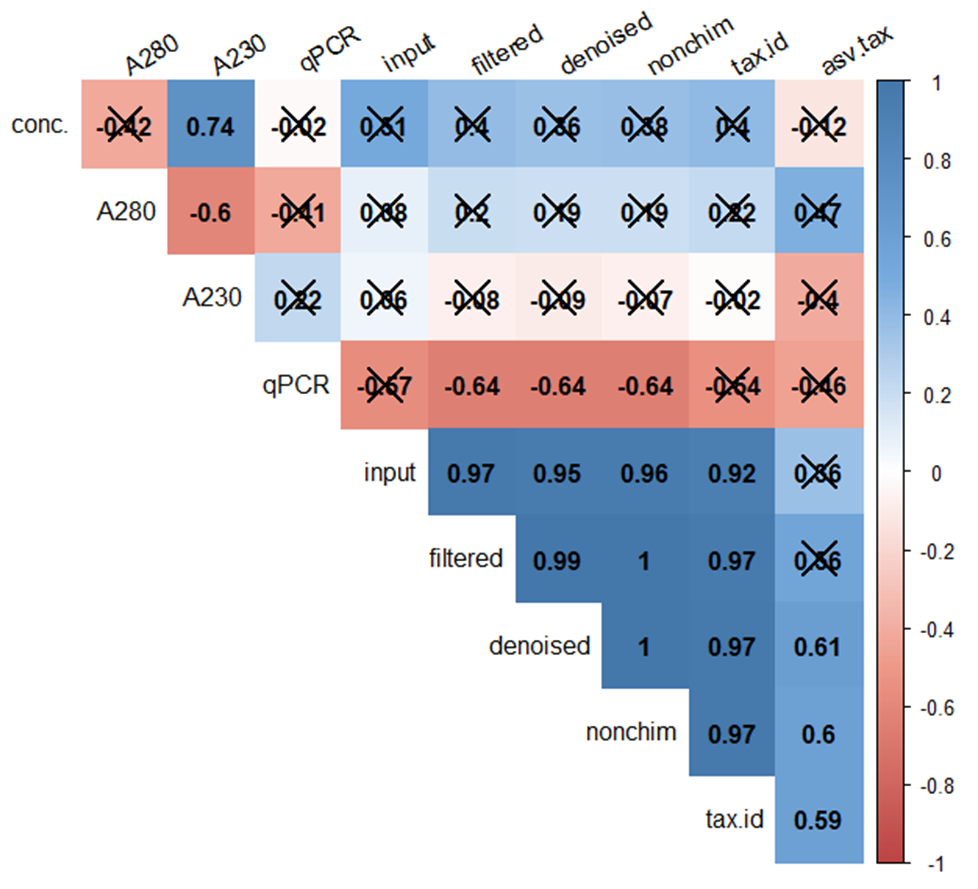
**

### **Figure S2.** Pearson’s correlation matrix on log-transformed values. Statically significant (p < 0.05) Pearson’s R values are highlighted. “conc.” Stands for extracted DNA (ng/μl); “A280” for 260/280 ratio of DNA purity; “A230” for 230/280 ratio of nucleic acid purity; “qPCR” for the PCR amplicon library concentration (nM); “input” for the raw HTS-reads; “filtered” for quality filtered reads; “denoised” for the denoised reads; “nonchim” for the non-chimeric reads; “tax.id” for the reads with taxonomic assignment; “asv.tax” for the ASV count with taxonomic assignment.


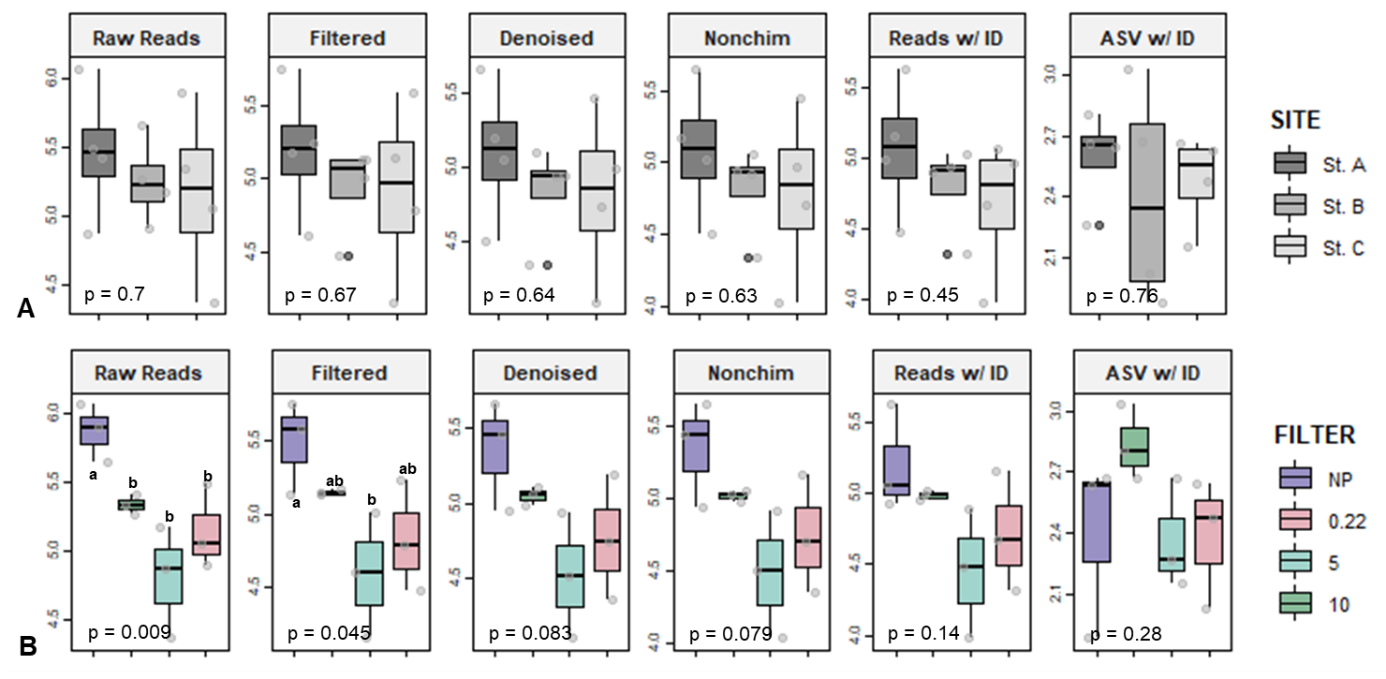


### **Figure S3.** Box-and-whisker plots of log-transformed read and amplicon sequence variant (ASV) abundance grouped by site **(A)** and filter **(B)**. Means with the same letter are not significantly different according to t-test at p < 0.05.


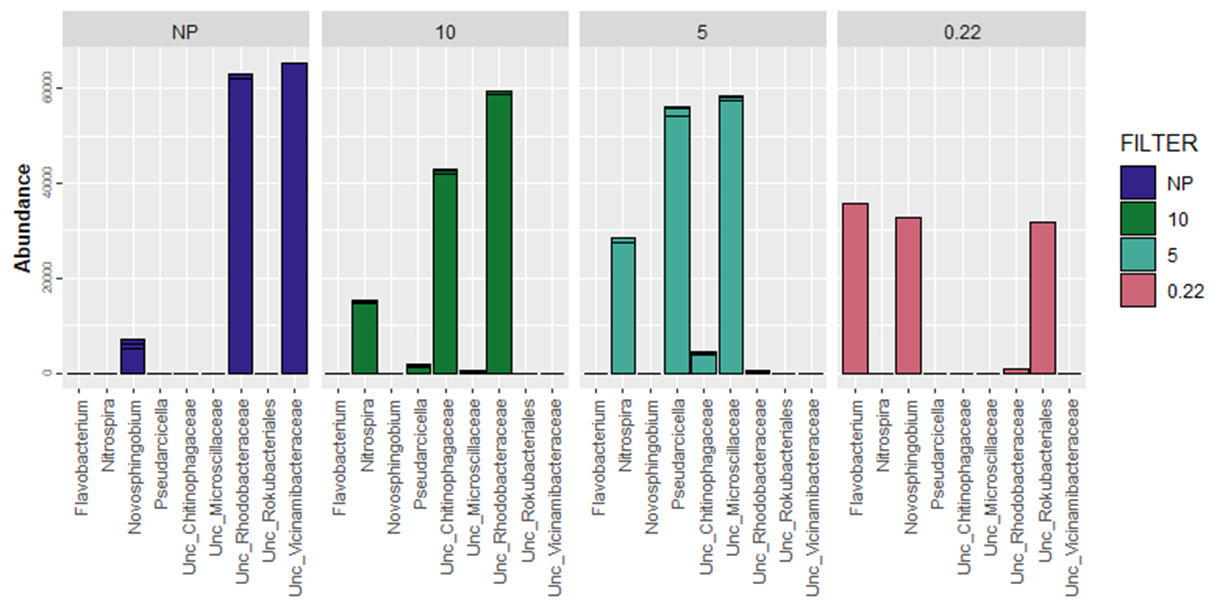


### **Figure S4.** Absolute abundance of the top 10 genera grouped by filter.


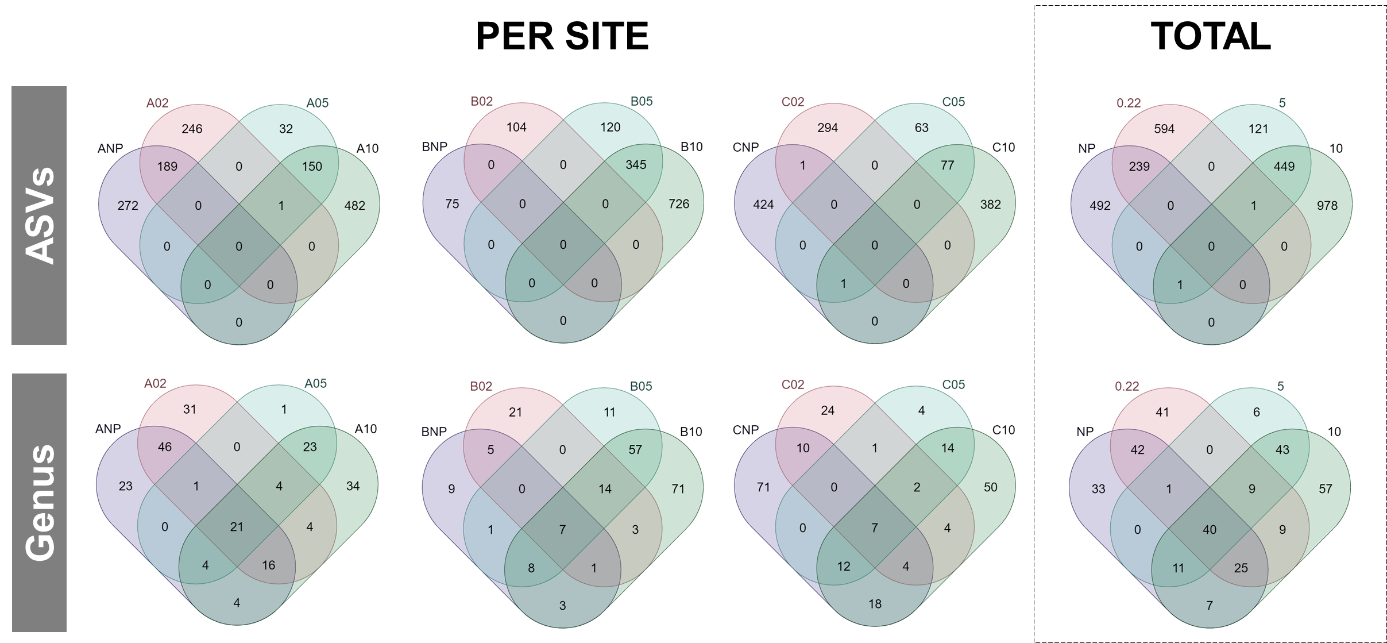


### **Figure S5.** Venn Diagrams showing shared and unique ASVs and genus between the filter types amongst sites.

**
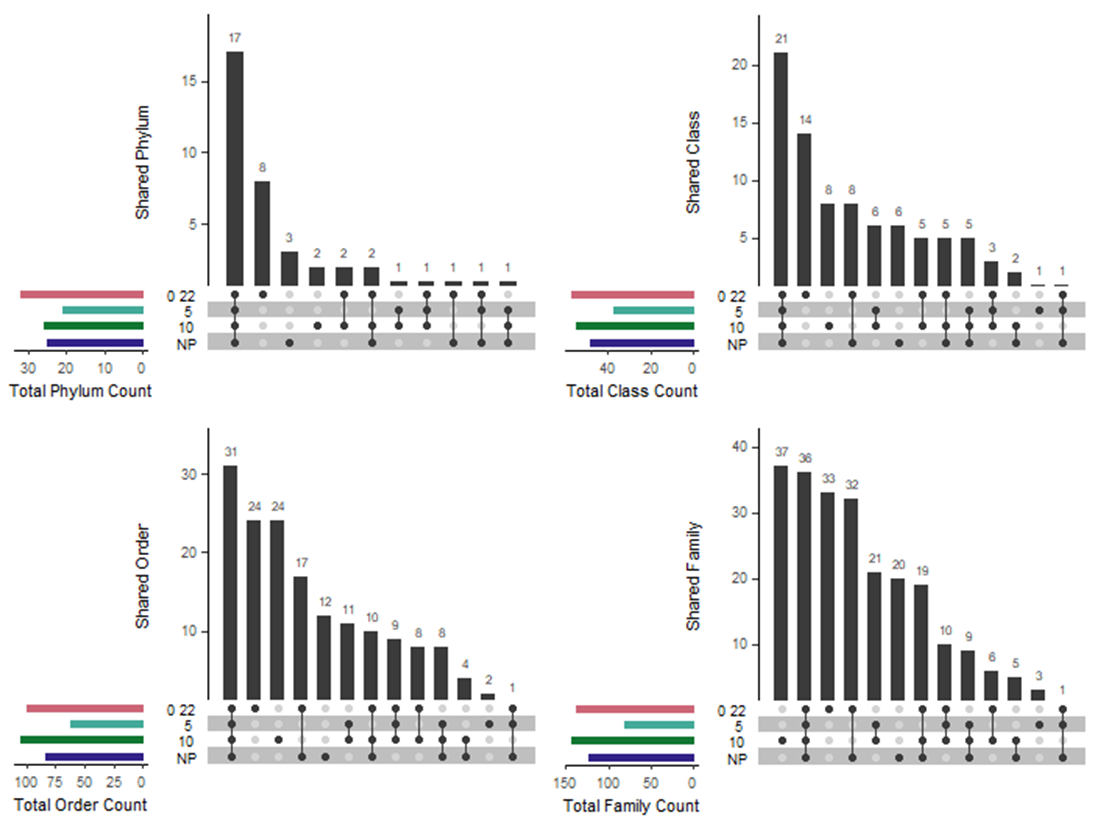
**

### **Figure S6.** UpSetR plots showing shared and unique taxa between the non-processed (NP) and filtered (10, 5, and 0.22 μm) sediment samples. The bars in the upset plot show the overlap between the indicated sample below.


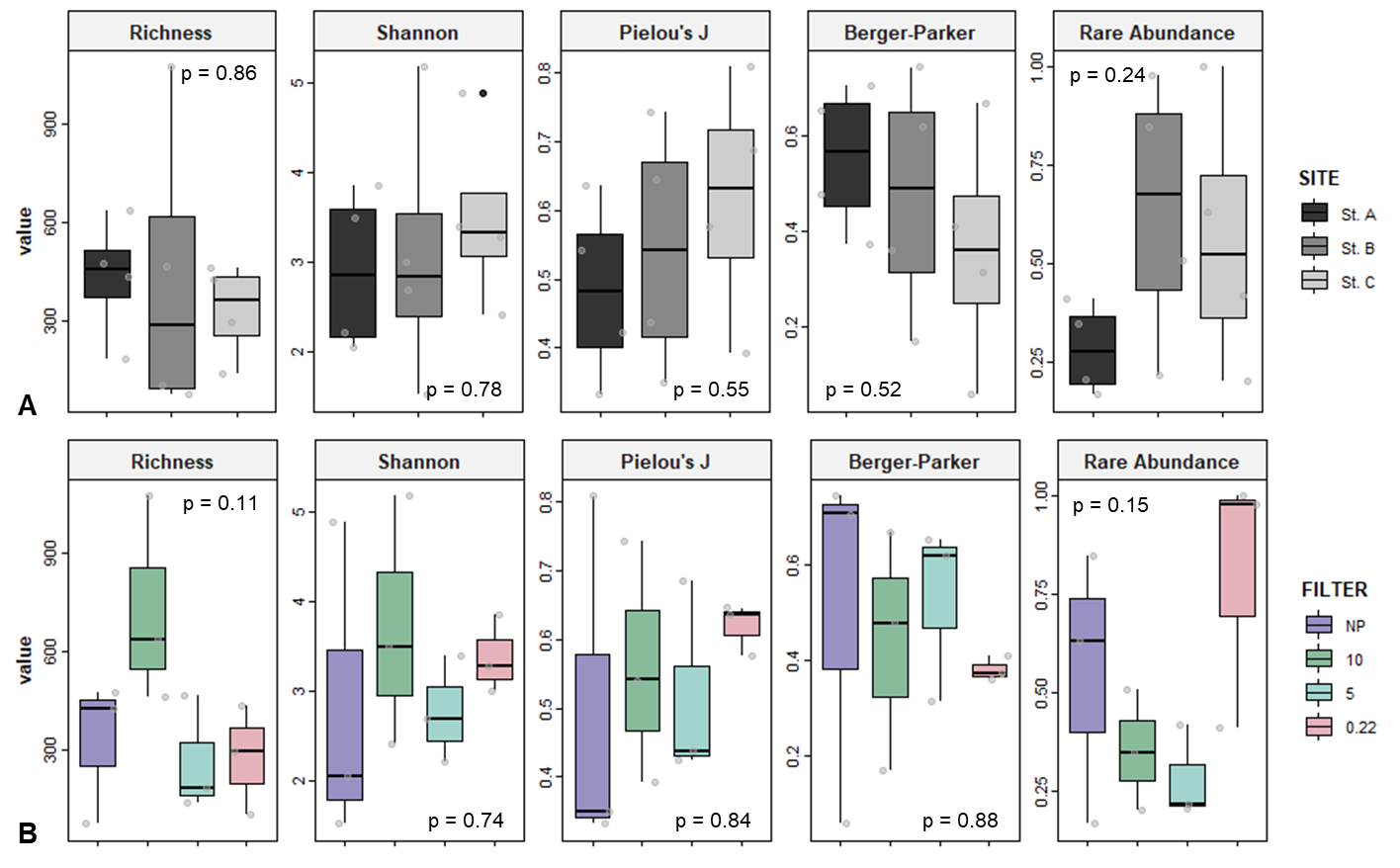


### **Figure S7.** Box-and-whisker plots of alpha diversity indices metrics comparing the samples by site **(A)**, and filter **(B)**.


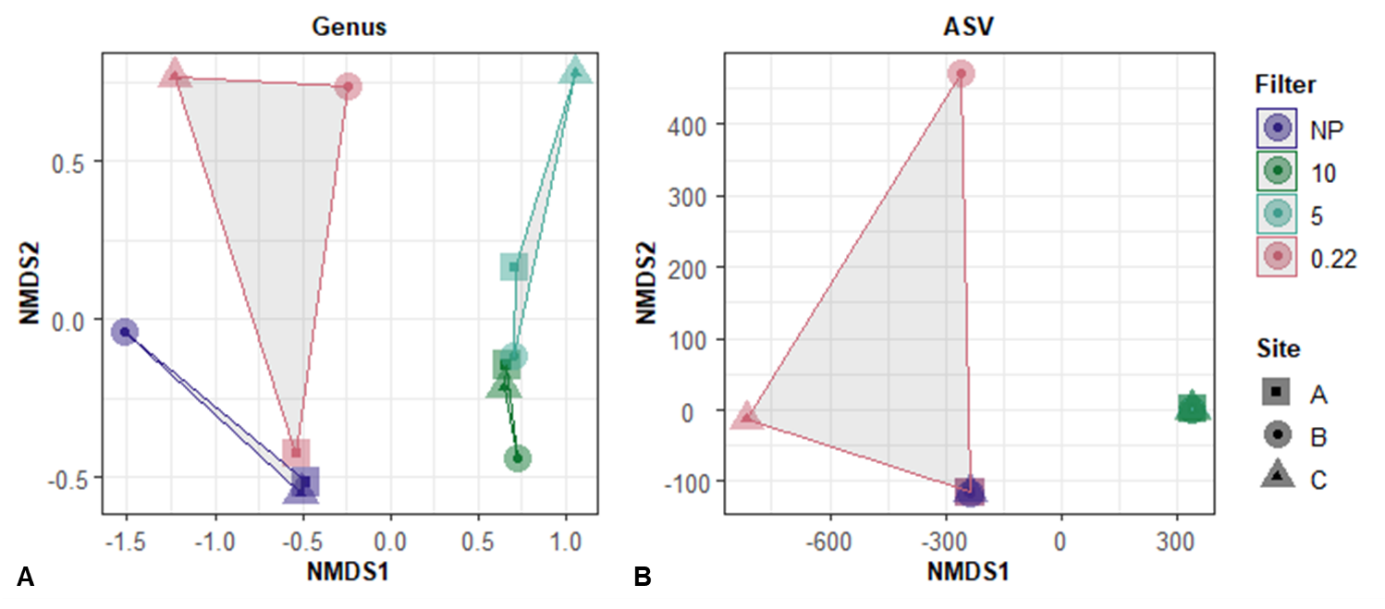


### **Figure S8.** Cluster analysis via non-metric multidimensional scaling (NMDS) based on Bray-Curtis dissimilarity showing microbial community composition the non-processed (NP), and sequential filtered (10, 5, and 0.22 μm) sediment samples for the genus **(A)** and ASV **(B)** datasets.
